# Adoptive T-cell immunotherapy for ganciclovir-resistant CMV disease after lung transplantation

**DOI:** 10.1038/cti.2015.5

**Published:** 2015-03-27

**Authors:** Chien-Li Holmes-Liew, Mark Holmes, Leone Beagley, Peter Hopkins, Daniel Chambers, Corey Smith, Rajiv Khanna

**Affiliations:** 1 South Australian Lung Transplant Unit, Department of Thoracic Medicine, Royal Adelaide Hospital, Adelaide, South Australia, Australia; 2 Faculty of Health Sciences, School of Medicine, University of Adelaide, Adelaide, South Australia, Australia; 3 QIMR Centre for Immunotherapy and Vaccine Development, QIMR Berghofer Medical Research Institute, Brisbane, Queensland, Australia; 4 Queensland Lung Transplant Service, The Prince Charles Hospital, Brisbane, Queensland, Australia; 5 School of medicine, The University of Queensland, Brisbane, Queensland, Australia

## Abstract

Infections with cytomegalovirus (CMV) can induce severe complications after solid organ transplantation (SOT). The prognosis for ganciclovir-resistant CMV infection and disease is particularly poor. Whereas adoptive transfer of CMV-specific T cells has emerged as a powerful tool in hematopoietic stem cell transplant patients, its translation into the SOT setting remains a significant challenge as underlying immunosuppression inhibits the virus-specific T-cell response *in vivo*. Here, we demonstrate successful expansion and adoptive transfer of autologous CMV-specific T cells from a seronegative recipient of a seropositive lung allograft with ganciclovir-resistant CMV disease, resulting in the long-term reconstitution of protective anti-viral immunity, CMV infection, disease-free survival and no allograft rejection.

The incidence of symptomatic cytomegalovirus (CMV) disease following solid organ transplantation (SOT) has decreased with the advent of ganciclovir, the mainstay of prophylactic, pre-emptive and treatment strategies.^1, 2^ However, ganciclovir-resistant CMV (GRCMV) disease poses major treatment difficulties, with significant morbidity and mortality due to end-organ effects and immunomodulation leading to bronchiolitis obliterans syndrome (BOS) and allograft loss.^3, 4, 5^ The incidence in lung transplant (LTx) recipients is up to 9%, the highest of any SOT,^6, 7^ with up to 17.6% of viraemic lung transplant recipients demonstrating resistance compared with 6.2% of viraemic renal transplant patients.^8^ Management includes cautious reduction in immunosuppression to allow immune reconstitution and activity against CMV,^7^ but this confers a risk of lung allograft rejection.^9^ Alternative treatments are limited: foscarnet is the first-line for the treatment of GRCMV.^10^ Although some patients treated with foscarnet have been shown to improve, the major problem in lung transplant recipients is significant nephrotoxicity.^7^ In addition, intravenous treatment is required, often with pre-hydration and reduction of other potentially nephrotoxic medications. This limits long-term use in treating and preventing recurrent disease. A further alternative, cidofovir, has no controlled studies but this agent is also highly nephrotoxic.^7, 11, 12^ Prognosis following GRCMV disease is therefore poor, with crude mortality ranging from 19 to 100%.^1, 8^ The study by Mitsani *et al.*^1^ reported 100% mortality as a direct result of CMV disease in four patients infected with GRCMV, despite the use of aggressive anti-viral regimens that included various combinations of foscarnet, cidofovir and CMV intravenous immunoglobulin (IVIg). These regimens are associated with significant toxicities and expense.^6^ Therefore, alternative effective therapeutic options are urgently required.

Immunologic control of CMV is complex and T-cell responses are critical. T-cell reactivity is directed towards a wide range of CMV antigens, and natural killer cells typically increase in response to viral replication.^13, 14^ Adoptive transfer of CMV-specific T-cell lines demonstrates promising results in hematopoietic stem cell transplantation (HSCT) recipients.^15, 16^ However, generation of specific T-cell lines *ex vivo* and their function *in vivo* is more complicated in SOT recipients and success has not been achieved previously—a single case report describes generation of CMV-specific T-cell lines from a patient with GRCMV disease, however the patient did not survive long term. ^17^ In HSCT, peripheral blood mononuclear cells (PBMCs) taken as starting material for CMV-specific T-cell generation are derived from healthy donors. In SOT, starting material must be recovered from immunosuppressed patients for autologous use.^17^ Following LTx, CMV disease is mainly due to seronegative recipients receiving seropositive donor organs, in contrast to post HSCT, when the majority is due to reactivation. Here, we present a case of successful adoptive transfer of autologous CMV-specific T cells to a LTx recipient with ganciclovir-resistant tissue-invasive CMV disease, resulting in uncomplicated long-term survival.

## Case report

**Figure 1:**
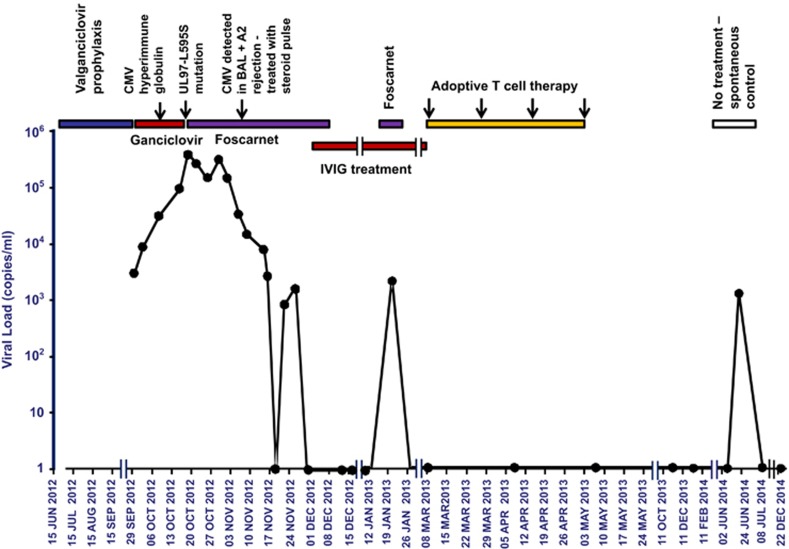
CMV replication before and after adoptive T-cell therapy. Timeline of viral replication as detected by a qualitative assay for CMV PCR in relation to anti-viral therapy and autologous adoptive T-cell transfer. The specific time point of detection of the UL97 mutation is also indicated in the graph.

A 41-year-old female (human leucocyte antigen (HLA)-A1, -A11, -B7, -B35, -Cw4, -Cw7, -DR1 and -DR14) received CMV mismatched (D+/R−) bilateral sequential LTx in June 2012 for cystic fibrosis. Immunosuppression was tacrolimus, prednisolone and azathioprine. CMV prophylaxis was CMV hyperimmune globulin and ganciclovir, followed by continuous valganciclovir 450 mg twice daily. The patient had mild renal impairment (estimated glomerular filtration rate of 46 ml min^−1^). Three months post transplant, she developed an asymptomatic increase in blood CMV PCR (DNAemia: 2769 DNA copies ml^−1^; Figure 1), new onset pancytopaenia (haemoglobin 97 g l^−1^, white cell count 3.64 × 10^9^ l^−1^, platelets 140 × 10^9^ l^−1^) and liver dysfunction (gamma glutamyl transferase (GGT) 175, alkaline phosphatase (ALP) 134, alanine aminotransferase (ALT) 79  and aspartate aminotransferase (AST) 69 U l^−1^) consistent with hepatitis. Despite intravenous ganciclovir and CMV hyperimmune globulin, the blood CMV PCR continued to rise. CMV PCR was also detected in bronchoalveolar lavage (BAL) fluid during bronchoscopy. Foscarnet was commenced. Leukopenia was minimised by cessation of azathioprine and trimethoprim/sulfamethoxazole. Tacrolimus was changed to everolimus and leflunomide added. CMV PCR peaked at 344 386 DNA copies ml^−1^ on day 18 post ganciclovir commencement (Figure 1). CMV gene results confirmed UL97 gene mutation L595S conferring ganciclovir resistance. Foscarnet was continued. Resolution of the pancytopaenia and hepatic dysfunction occurred, while CMV PCR fluctuated and gradually reduced to undetectable levels after 6 weeks of continuous treatment with foscarnet and reduced immunosuppression. Surveillance transbronchial biopsies showed mild (A2) acute cellular rejection, treated with methylprednisolone and oral steroids and fortnightly IVIg. After 2 weeks of undetectable CMV PCR levels, foscarnet was ceased. Two weeks later, the patient had another asymptomatic rise in CMV PCR (2059 DNA copies ml^−1^) requiring a further 2 weeks of foscarnet, which reduced the CMV PCR back to undetectable (Figure 1). The patient continued IVIg fortnightly until T-cell therapy was commenced 5 months after the initial presentation.

## Treatment

**Figure 2:**
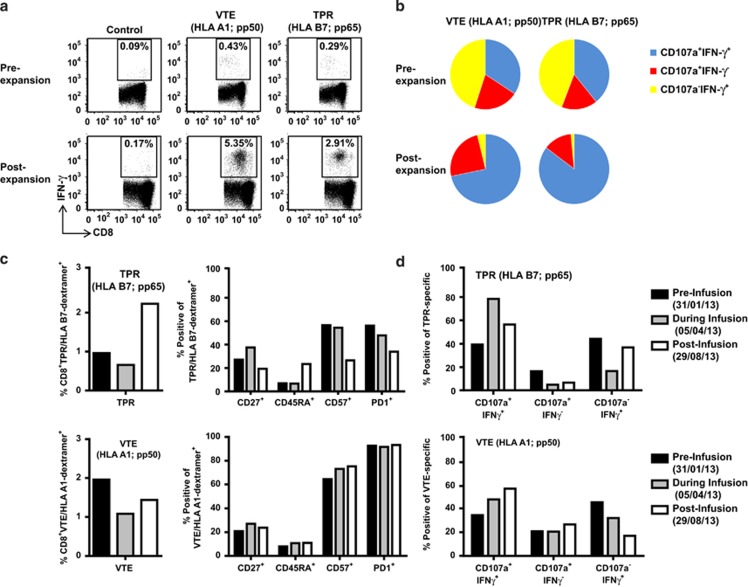
Functional characterisation of CMV-specific T cells. (**a**) PBMC and *in vitro* expanded T cells were stimulated for 5 h with cognate peptide in the presence of brefeldin A, then assessed for IFN-γ expression. Data presented in each subpanel indicates the percentage of peptide-specific T cells producing IFN-γ. (**b**) PBMC and *in vitro* expanded T cells were stimulated for 5 h with cognate peptide in the presence of brefeldin A and monensin, then assessed for IFN-γ expression and degranulation using surface CD107a expression. Data in each pie chart represents the proportion of VTE or TPR specific T cells expressing CD107a and IFN-γ (blue) CD107a alone (red) or IFN-γ alone (yellow). (**c**) PBMC were labelled with the HLA-B7/TPR (pp65) or HLA-A1/VTE (pp50) dextramer then assessed for surface expression of CD27, CD45RA, CD57 and PD1. Data in the left sub-panels represents the frequency of TPR (pp65) or VTE (pp50)-specific CD8^+^ T cells. Data in the right sub-panels represents the proportion of TPR- or VTE-specific CD8^+^ T cells expressing each phenotypic marker. (**d**) PBMC were stimulated for 5 h with TPR (pp65) or VTE (pp50) peptide in the presence of brefeldin A and monensin, then assessed for IFN-γ expression and degranulation using surface CD107a expression.

We obtained approval from the ethics committee to expand and infuse autologous CMV-specific T cells generated from the patient as outlined in the Methods section below. On the basis of the HLA type of the patient, CMV-specific immunodominant peptide epitopes were selected for T-cell expansion.^18^ A total of 12 × 10^7^ T cells were expanded and equally divided into four vials. The dose selected per vial was based upon the total number of cells that were manufactured. Phenotypic analysis showed that the expanded cells were predominantly T cells CD3^+^ (82.6%), including both CD8^+^ (73.8%) and CD4^+^ (14%) T cells. T cells specific for all of the peptide epitopes used in the study could be detected following expansion. Analysis using an intracellular interferon (IFN)-γ assay revealed an increase in the proportion of CMV-specific T cells directed towards the HLA-A1-restricted VTEHDTLLY (5.35%), HLA-A1-restricted YSEHPTFTSQY (2.26%), HLA-B7-restricted TPRVTGGGAM (2.91%), HLA-B7-restricted RPHERNGFTVL (0.46%), HLA-B35-restricted FPTKDVAL (0.35%), and HLA-B35-restricted IPSINVHHY (0.35%) peptide epitopes. Representative data from the dominant pp50 encoded, VTEHDTLLY (referred to as VTE) and the pp65 encoded epitope, TPRVTGGGAM (referred to as TPR) are shown in Figure 2a. These *in vitro* expanded T cells also displayed an increase in cytolytic potential, characterised by an increase in the proportion of IFN-γ^+^CD107a^+^ cells (Figure 2b).

The patient received four infusions of autologous adoptive T-cell immunotherapy. No adverse events occurred. *Ex vivo* virus-specific T-cell immunity in the peripheral blood was monitored using MHC-multimer analysis, surface receptor phenotyping and polyfunctional cytokine analysis. Althogh there was an increase in the frequency of pp65-specific T cells in the peripheral blood, pp50-specific T-cell numbers decreased following the completion of adoptive immunotherapy. *Ex vivo* surface phenotypic analyses revealed a reduction in the proportion of CD57^+^ and PD1^+^ pp65-specific T cells post infusion, but no change in pp50-specific T cells (Figure 2c). However, intracellular cytokine analyses showed that both pp65 and pp50-specific T-cells displayed an increased proportion of IFN-γ^+^CD107a^+^ T cells during and post infusion (Figure 2d).

The patient's usual immunosuppression regime was resumed and CMV PCR remained negative in plasma, BAL and transbronchial biopsies for 16 months, at which point an asymptomatic increase in CMV PCR occurred. This became undetectable rapidly with no medical intervention or alteration in treatment. At 20 months post treatment, the CMV PCR remains undetectable, hepatic and bone marrow function remains normal and there has been no evidence of acute rejection on transbronchial biopsies, or BOS on serial lung function measurements.

## Comment

In this report, we describe the successful treatment of primary and persistent GRCMV infection post LTx using adoptive T-cell immunotherapy. After persistently elevated blood CMV PCR levels, evidence of hepatitis as end-organ disease despite prolonged treatment with foscarnet and recurrence of detectable PCR when foscarnet was ceased briefly, the patient immediately became completely CMV PCR negative and has remained controlled for 20 months post-T-cell therapy, without any other pharmacological treatment, while on standard immunosuppression. Other treatment options were unacceptable: foscarnet was not feasible for long term due to renal impairment. Without adequate control of CMV infection, long-term reduced immunosuppression would have been required, potentially leading to further episodes of acute rejection, reduced allograft function and worse survival. Were the patient to survive, high medical costs and poor quality of life would ensue.

To date, there are few reports of adoptive T-cell immunotherapy for treatment of ganciclovir-resistant CMV infection or disease following SOT. In the only report following LTx,^17^ CMV disease responded to immunotherapy initially but recurred after 4 weeks and viral load stayed despite a second infusion of CMV-specific T cells. After 6 weeks, the patient developed cellular rejection and was treated with steroids, leading to an increase in peripheral CMV viral load. The patient died from histologically proven graft failure 2 weeks later. It is not clear why the transferred T cells did not totally clear the virus from the blood, and it was not possible to exclude an association between rejection and the T-cell application, and high caution was recommended. Our experience shows that established CMV infection can be cleared, and demonstrates success in utilisation of adoptive T-cell immunotherapy to treat GRCMV disease post LTx, in the presence of immunosuppression, without complications. Although it is possible that partial immune restoration may have also contributed towards viral control, the improved cytolytic potential in both pp65- and pp50-specific T cells following infusion suggests that adoptive therapy may have contributed to the improved immune control of CMV. Although four doses of T cells were administered, evidence of persistent viral reactivation was not evident following administration of the first vial, suggesting that a single dose of T cells may have been adequate to control infection. In addition, the persistent episodes of relapse before therapy and cessation of episodes immediately following treatment, as well as control of a late asymptomatic rise in CMV PCR without anti-CMV treatment shows that the patient achieved stable immune reconstitution which was able to control the viral reactivation, representing proof of concept for T-cell immunotherapy. The data are relevant beyond this case, highlighting the exciting potential of this approach as a new treatment option where no adequate treatment strategies previously existed. Future research will focus on developing the therapy for larger patient numbers.

## Methods

### Generation of CMV-specific T cells for adoptive immunotherapy

To expand CMV-specific T cells, PBMC were stimulated with autologous PBMC coated with HLA class I-restricted CMV peptide epitopes VTEHDTLLY, YSEHPTFTSQY, TPRVTGGGAM, RPHERNGFTVL, FPTKDVAL, and IPSINVHHY; as outlined previously.^19^ The peptides were selected based on the HLA class I typing of the patient. After 14 days, these T cells were harvested and tested for microbial contamination. T cells were prepared for intravenous infusion by washing twice in normal saline then resuspended in 20 ml normal saline for infusion.

### Monitoring of CMV-specific T-cell immunity

CMV-specific T cells before and after adoptive therapy were assessed for cellular phenotype and T-cell functionality. Intracellular cytokine flow cytometer assays, on PBMC or *in vitro* expanded T cells were performed as previously described.^19^ CD8^+^ T cells were assessed for cytolytic potential using fluorescein isothiocyanate (FITC)-conjugated anti-CD107a (BD Biosciences, Franklin Lakes, NJ, USA) and for intracellular cytokine using PE-conjugated anti-IFN-γ or AlexaFluor700-conjugated anti-IFN-γ (BD Biosciences). To assess the phenotype of HLA-B7/TPRVTGGGAM and HLA-A1/VTEHDTLLY MHC class I dextramer (Immudex, Copenhagen, Denmark) specific cells, PBMC was stained with PerCP-Cy5.5-conjugated anti-CD8 (eBioscience, San Diego, CA, USA), PE-Cy7-conjugated anti-CD4 (BD Biosciences), V450-conjugated anti-CD27 (BD Biosciences), FITC-conjugated anti-CD45RA (BD Biosciences), PE-conjugated anti-PD1 (BD Biosciences) and biotin-conjugated anti-CD57 (BD Biosciences). All cell acquisitions were performed on a BD Fortessa with FACS Diva software, and post-acquisition analysis was performed using FlowJo software (TreeStar, Ashland, OR, USA).

### CMV load monitoring

CMV DNA was extracted from plasma or serum samples and quantitation of CMV viral load was performed by the importation of a standard curve created using the Acrometrix CMV standard panel (Life Technologies, Carlsbad, CA, USA) according to manufacturers' instructions. Each concentration was tested triplicate. Inter-run variance was corrected for by the inclusion of calibrators and controls in each run.
